# Tiotropium respimat^®^ add-on therapy reduces airflow obstruction in patients with symptomatic moderate asthma, independent of T_H_2 inflammatory status

**DOI:** 10.1186/1710-1492-10-S2-A52

**Published:** 2014-12-18

**Authors:** WH Yang, T Casale, ED Bateman, R Dahl, E Pizzichini, M Vandewalker, JC Virchow, M Engel, PM Moroni-Zentgraf, H Schmidt, HAM Kerstjens

**Affiliations:** 1 Allergy & Asthma Research Centre, Ottawa, Canada, K1Y 4G2; 2 Division of Allergy and Immunology, Creighton University, Omaha, NE, USA; 3 Department of Medicine, University of Cape Town, Cape Town, South Africa; 4 Aarhus University Hospital, Aarhus, Denmark; 5 NUPAIVA (Asthma Research Centre), Universidade Federal de Santa Catarina, Florianópolis, Brazil; 6 Clinical Research of the Ozarks, Columbia, MO, USA; 7 Department of Pulmonology, Intensive Care Medicine, Zentrum für Innere Medizin, Klinik I, University Clinic Rostock, Rostock, Germany; 8 TA Respiratory Diseases, Boehringer Ingelheim Pharma GmbH & Co. KG, Ingelheim am Rhein, Germany; 9 Boehringer Ingelheim Pharma GmbH & Co. KG, Biberach an der Riss, Germany; 10 Department of Pulmonary Medicine, University Medical Center Groningen, University of Groningen, Groningen, The Netherlands

## Rationale

In patients with symptomatic asthma receiving ICS or ICS+LABA, Phase III studies have demonstrated improved lung function with tiotropium Respimat^®^, a once-daily long-acting anticholinergic bronchodilator. The efficacy of some treatments (eg ICS and omalizumab), appears higher in T_H_2-high phenotypes, but no specific treatments are available that work equally well in both T_H_2-high and T_H_2-low phenotypes. We explored whether T_H_2 biomarker status influenced responses to tiotropium in patients with moderate symptomatic asthma.

## Methods

In two replicate Phase III, randomized, double-blind, placebo-controlled, parallel-group trials (NCT01172808/NCT01172821), patients with moderate symptomatic asthma, using medium-dose ICS (400-800 µg budesonide equivalent), were administered once-daily tiotropium Respimat^®^ 5 µg or 2.5 µg, placebo, or salmeterol (active comparator without inferential analysis). Co-primary endpoints included peak and trough FEV_1_ response (difference from baseline) at 24 weeks. Pre-planned analyses (pooled population) were performed in T_H_2-high and T_H_2-low subgroups defined at baseline as total serum IgE ≤ or >430 µg/L) or blood eosinophils ≤ or >0.6×10^9^/L.

## Results

Of 1545 patients in the full analysis set who received tiotropium or placebo, 915/1455 were reported with IgE >430 µg/L and 300/1461 with an eosinophil count of >0.6×10^9^/L. Peak FEV_1_ improved with tiotropium versus placebo, independent of IgE (p<0.0001 both doses) and eosinophil count (p<0.0001 both doses). Trough FEV_1_ also improved with tiotropium versus placebo, irrespective of IgE (p<0.0001 both doses) and eosinophil count (p<0.005 both doses).

## Conclusions

Once-daily tiotropium Respimat^®^ as add-on to ICS reduces airflow obstruction in patients with moderate symptomatic asthma, independent of T_H_2 phenotype, and thus may potentially provide an important therapeutic option.

## Funding source

Study supported by Boehringer Ingelheim. Previously presented at AAAAI 2014 in San Diego, CA, USA.

